# Geographic boundaries and natural history notes of the microendemic endangered frog *Eupsophus migueli* Formas, 1977 (Alsodidae) in the Mahuidanche Range, southern Chile

**DOI:** 10.3897/zookeys.929.35984

**Published:** 2020-04-22

**Authors:** César C. Cuevas, Rocío Sanhueza

**Affiliations:** 1 Departamento de Ciencias Biológicas y Químicas, Universidad Católica de Temuco, Rudecindo Ortega, Casilla 15-D, Temuco, Chile Universidad Católica de Temuco Rudecindo Ortega Chile; 2 Riparia LTDA, Valdivia, Chile Unaffiliated Valdivia Chile

**Keywords:** conservation, frogs, microendemism, new geographic records, temperate forest

## Abstract

*Eupsophus migueli* is considered a microendemic endangered species inhabiting the temperate *Nothofagus* forests of the Mahuidanche Range of southern Chile. However, this categorization is based on scarce data about its distribution and natural history. In order to assess these parameters, this article reports new geographic records obtained through intensive fieldwork between 2011 and 2016. Considering this, an updated distribution map for *E. migueli* is proposed, and new data about natural history traits and habitat use are provided. The information obtained in this study is discussed considering the zoogeographical importance of *E. migueli*, and confirms the species IUCN conservation status.

## Abstract (es)

*Eupsophus migueli* está considerada como una especie microendémica en peligro de extinción que habita los bosques templados de *Nothofagus* de la Cordillera de Mahuidanche, sur de Chile. Sin embargo, esta categorización está basada en poca información acerca de su distribución e historia natural. Con el fin de evaluar ambos parámetros, en este artículo se reportan nuevos registros geográficos, obtenidos de manera intensiva entre los años 2011 y 2016. De esta forma, se propone un mapa de distribución actualizado para *E. migueli*, y se presentan nuevos datos sobre los rasgos de historia natural y sobre el uso del hábitat. Los nuevos antecedentes se discuten en referencia a la importancia zoogeográfica de *E. migueli*, y reafirman su categoría de conservación.

## Introduction

Species of the genus *Eupsophus* (Feitzinger, 1843) are endemic to the temperate *Nothofagus* forests of central and southern Chile, and part of Argentina.

These species have a wide distribution range, being found from the remnant Maulino forest in the north (35°50'1.92"S, 72°30'36.31"W), to Isla Wellington in the south (46°25'S, 72°04'W) (Núñez et al. 2012). The extent of occurrence of the genus is about 191,978 km², however, microendemic conditions have been documented for *E. septentrionalis* (Ibarra-Vidal et al. 2004, Veloso et al. 2005), *E. insularis* (Formas and Vera 1982), *E. contulmoensis* (Ortiz et al. 1989), *E. nahuelbutensis* (Ortiz and Ibarra-Vidal 1992), *E. altor* (Núñez et al. 2012) and *E. migueli* (Formas 1979, Méndez et al. 2005). Here, we discuss the case of the microendemic frog *Eupsophus migueli* Formas, 1977.

**Figure 1.**
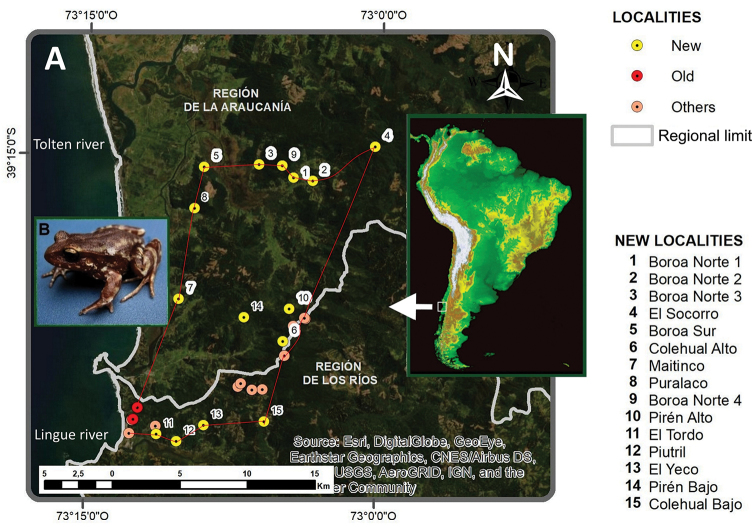
**A** Distribution map of *Eupsophus migueli*. The red polygon corresponds to an updated distribution area of *E. migueli*, and it is formed by georeferenced landmarks including new records (this paper), old records (including the type locality, Mehuín, and nearby localities Queule and Pichicuyín) and other documented points (Méndez et al. 2005, Contreras 2014, Miranda 2015) **B** specimen of *E. migueli* from Colehual Alto.

*Eupsophus migueli* has been described in the Coastal Range of southern Chile. For a long time, its known distribution range was restricted only to a few sites neighbouring the type locality (Mehuín, 39°25'42.46"S, 73°12'42.35"W), covering an area of no more than 2.4 km^2^. Later, its northern limit was located near Queule (39°22'S, 73°08'W; Méndez et al. 2005), seven kilometers north of the type locality (see Fig. 1). Although, Correa et al. (2017) synonymized *E. altor* with *E. migueli* extending the range of *E. migueli* south to the Lingue River, Suárez-Villouta et al. (2019) hypothesised that both are good species restoring the previous taxonomic situation. Bradford and Jaffré (2004) suggested that highly restricted endemism’s, like the one exhibited by *E. migueli*, are typically attributed to a complex interplay of climatic, topographic, and geologic diversity, derived from environments with unique geography and severe levels of native habitat fragmentation. Although the microendemic status of *E. migueli* seems plausible in accordance with the description provided by Bradford and Jaffré (2004), intensive geographic studies have not been feasible since the species’ description. Hence, we hypothesize that the highly restricted distribution of *E. migueli* is actually due to insufficient field sampling. Therefore, based on the compilation of sufficient data, its status as a microendemic species could be questioned. Consequently, the present study describes results of an exhaustive fieldwork conducted between 2011 and 2016, eastward and northward from the type locality of *E. migueli*. Finally, we comment on the taxonomic status of *E. migueli* and, on the base of all records available, we reassess and confirm its current conservation status according to the Chilean Regulation for Species Classification (RCE in spanish) and the International Union for Conservation of Nature (IUCN) Red List criteria.

## Materials and methods

### Study area

The study took place in a section of the Chilean Coastal Range known by the locals as Cordillera de Mahuidanche (about 55 km long and 20 km wide, with a maximum altitude 715 m a.s.l.). This mountain range is located in southern Chile between the mouths of Toltén (39°01'S, 73°06'W, Cautín Province) and Valdivia rivers (39°52'S, 73°23'W, Valdivia Province). The surveyed area comprises a polygon extending from south to north from the northern bank of the Lingue River (Mariquina, Los Ríos Region) to the southern bank of the Toltén River (Toltén, Araucanía Region); and from west to east, from the slopes of the Mahuidanche mountain (Lastarria, Araucanía Region), to the border between La Araucanía and Los Ríos Regions. The study area covered about 833.331 km² (Fig. 1).

The vegetation of the study area is characterized by the presence of the Laurifolia plant community, typically associated to the Valdivian rain forest (Gajardo 1993). Such community has been modified by human action, appearing today mostly forming remnants of mixed relict vegetation, belonging primarily to the associations of roble-laurel-lingue and temo-pitra in hillocks and low-lying areas, respectively (Hauenstein et al. 2002). Parts of these native forest remnants have been considered a priority for the conservation of biodiversity (CONAMA 2002).

### Taxonomic criteria

Formas (1977), based on external morphometric, morphological qualitative characters, and chromosomic attributes, described *E. migueli*. Formas (1989) divided *Eupsophus* in two groups: “roseus” group (species presenting chromosome formula 2n = 30, snout–vent length < 6 cm; *E. altor*, *E. calcaratus*, *E. contulmoensis*, *E. insularis*, *E. migueli*, *E. nahuelbutensis*, *E. roseus* and *E. septentrionalis*), and the “vertebralis” group (species with 2n = 28, adult snout- vent length > 6 cm; *E. vertebralis* and *E. emiliopugini*). Correa et al. (2017) hypothesized that several species of the *Eupsophus* “roseus” group were synonyms, decreasing their number from eight to four. Among these, *E. altor* was synonymized with *E. migueli* increasing the range of the latter. Correa et al. (2017) downplay some diagnostic characters that identify *E. altor* (Núñez et al. 2012). They argue that the karyotype, the maximum frequency of the spectral elements of the mating call, the morphometric analyses, and the lack of knowledge about the reproductive cycle and the larva of *E. migueli*, do not allow the differentiation of two species (see Núñez et al. 2012). Subsequently, Suárez-Villouta et al. (2019) based on a new molecular analysis (including coalescence tests) concluded that the eight species recognized in the “roseus” group before the work of Correa et al. (2017) were valid, which was corroborated by Correa and Durán (2019). In summary, in this work we consider the “roseus” group to contain eight described species. Thus, in order to avoid confusion between the species of the “roseus” group potentially present in the study area, specific determination of frogs in the field followed the original diagnosis provided by Formas (1977) for *E. migueli*, Formas and Vera (1982) for *E. roseus* and *E. calcaratus* and by Núñez et al. (2012) for *E. altor*.

For genus nomenclature we followed Feitzinger (1843) and Pyron and Wiens (2011) for the family taxonomy.

### Sampling

Systematic and standardized searches were conducted by applying Visual Encounter Surveys and time constrained search methods (Bury and Raphael 1983, Crump and Scott 1994) on transects of 100 m in length with a fixed width of 3 m. Searches were conducted in terrestrial environments within remnants of native secondary and mature forest, and exotic plantations. We focussed on adult frogs, performing a thorough visual examination of the area, and paying special attention to any possible shelters under rocks, logs, branches and leaves.

Calculations of two important IUCN criteria AOO (area of occupancy) and EOO (extent of occurrence) in km^2^, were done with GeoCAT (Geospatial Conservation Assessment) software (Bachman et al. 2011). The AOO calculation was based on a default IUCN value of 2 km.

### Specimens

Each collected specimen was placed into an individual plastic box, labelled with a characteristic colour and number. Collection sites were georeferenced and marked with the same colour of the respective specimen. Specimens were then transported to a near workstation, where they were measured with a calliper to the nearest 0.1 cm and photographed. Subsequently, each specimen was released at the exact place of capture. This study was conducted in strict accordance with published biosafety protocols in order to protect the health of amphibian populations, which involved the use of nitrile gloves and Virkon to disinfect any material used during the surveys (Soto-Azat et al. 2013). Sampling permits were granted by the Agricultural and Livestock Service (SAG) according to resolutions No. 4494/2012 and 5088/2016 to C. Cuevas.

**Figure 2.**
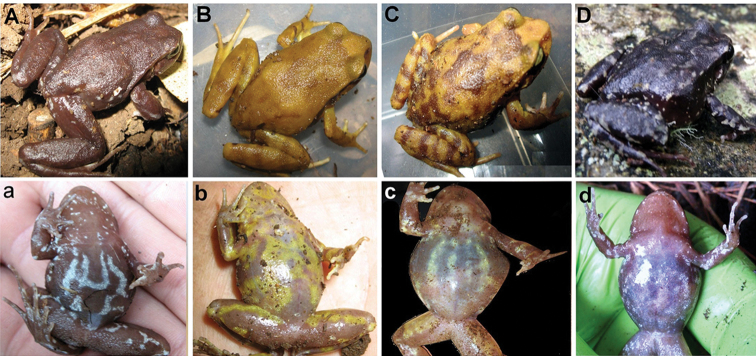
Dorsal (capitals) and ventral (lowercase) patterns of pigmentation in *Eupsophus migueli* from different localities along its distribution range. **A** Dorsal pattern of dark brown (Socorro) **a** ventral white with longitudinal spots (El Socorro, Boroa Norte) **B** dorsal yellow pattern and **b** belly with yellow crosslinks (El Socorro) **C** dorsal pattern yellow with brown spots and **c** belly with yellow longitudinal spots (El Socorro, Boroa Norte) **D** dorsal pattern dark brown and **d** belly with whitish faded spots. All specimens were adults ranging in size from 4 to 5 cm.

As no specimens were taken away from their habitat, the taxonomic determination of specimens as *E. migueli* in all the new localities reported in this work was obtained through photography and analysis of live specimens (froglets and adults) during field trips conducted between 2011–2012 (August to November both years) and 2014–2016 (January to December both years). In one of the field trips, we were accompanied by the authority of the species *E. migueli* (Dr R. Formas), who confirmed the taxonomic determination of the specimens. Although some morphological variation was detected (see Fig. 2), all specimens were identified as *E. migueli* by the following combination of external characters: yellow iris and belly with whitish reticulations on a wine base (Formas 1977).

## Results and discussion

New locality records reported here are located north of the town of Queule (Región de La Araucanía, Coastal Range), and south east of the village of Mehuín (Región de Los Ríos) (Table 1, Fig. 1). Some of the new localities, were detected during field trips conducted in 2007 by one of us (R. Sanhueza) and revisited for this study.

Early records considered herein include: Quebrada Casanova (Mehuín, type locality), Pichicuyín, and Queule located northern of Lingue River (Méndez et al. 2005). We also included others records reported by Contreras (2014) and Miranda (2015) (see map in Fig. 1). The taxonomic criteria used in this work to determine the specimens of *E. migueli* in the field, agrees with the results obtained by Suárez-Villouta et al. (2019), recognizing this taxon as a specific taxonomic unit different from *E. altor*. In this work we assume the conclusions of Suárez-Villouta et al. (2019), therefore, we consider the distribution of *E. migueli* from the Lingue River to the north. Consequently, all data available allows for the establishment of a broader distribution range for *E. migueli*. These new records reach ≈ 200 km^2^ extending its range to the north, northeast and east of the previously known range of the species. Old and new localities (names, coordinates and additional data) are shown in Table 1.

**Table 1.** New localities, coordinates and data (year, month, number (*N*) and mode of detection: calling (c) and/or visual (v)) of *E. migueli* specimens recorded during fieldwork reported in this paper.

ID	Locality	Coordinates	High	Year (month)	*N*	Detection
1	Boroa N 1	39°15'14.87"S, 73°06'16.91"W	4	2007–2015 (Jan)	20/1	v/c
2	Boroa N 2	39°15'44.72"S, 73°04’29.82"W	66	2007 (May)	10	v
3	Boroa N 3	39°14'57.44”S, 73°05'28.45"W	274	2007 (May)–2015 (Jan, Mar)	5/13	v
4	El Socorro	39°12'57.52"S, 73°00'45.32"W	450	2007 (Feb)–2015 (Mar)	10/7	v/c
5	Boroa Sur	39°15'04.15"S, 73°09'08.07"W	37	2011 (Sep)	5	c
6	Colehual Alto	39°22'28.83"S, 73°04'53.52"W	650	2011 (Sep)	13	c
7	Maitinco	39°20'45.23"S, 73°10'16.27"W	23	2011 (Nov)	3	v/c
8	Puralaco	39°17'04.83"S, 73°09'32.75"W	19	2012 (Aug)	5	c
9	Boroa N 4	39°15'17.23"S, 73°06'01.53"W	53	2015 (Apr)	4	v/c
10	Pirén Alto	39°21'38.91"S, 73°04'38.69"W	540	2015 (Apr)	7	v
11	El Tordo	39°26'07.55"S, 73°11'17.09"W	30	2015 (Dec)	2	c
12	Piutril	39°26'24.29"S, 73°10'14.37"W	40	2015 (Dec)	1	v
13	El Yeco	39°25'44.44"S, 73°08'52.00"W	86	2015 (Dec)	2	v
14	Pirén Bajo	39°21'24.12"S, 73°06'52.80"W	450	2016 (Dec)	3	v
15	Colehual Bajo	39°25'32.88"S, 73°05'44.97"W	282	2016 (Dec)	2	v

### External variation

Some froglets specimens detected from Boroa Norte in 2007 (Espinoza 2008, Sanhueza 2013) were misidentified as other *Eupsophus* spp., due to their iris colour and a very polymorphic dorsal pattern of pigmentation. In fact, the yellow colour of the iris of *E. migueli* is also shared with *E. altor* and *E. calcaratus*, however, their belly in a wine like pattern (Formas 1977) is quite different from both *E. calcaratus* and *E. altor*. In *E. altor* and *E. calcaratus* the belly is translucent (skin colour in *E. altor*), in addition, *E. calcaratus* exhibits a very characteristic black ribbon on the flanks of the head extending from the nares to the insertion of the forearms (Formas and Vera 1982). Moreover, *E. calcaratus* has never been reported north of the Valdivia River in Los Ríos Region (70 km south). Méndez et al. (2005) reported a population from the Mississippi locality south of the Lingue River (near Mehuín, type locality of *E. migueli*), however, the authors may have mistakenly identified specimens of *E. altor* as *E. calcaratus*. The current study confirms that identification as erroneous, since one of us (C. Cuevas) identified an *E. altor* specimen at the border of the Quilatrayen Stream (39°27'47.98"S, 73°9'18.68"W) (south of Lingue River) in December of 2015. This place is located 7 km north-east from the site reported by Méndez et al. (2005).

Specimens detected during our field work present intermediate patterns of pigmentation on the belly, as well as on the dorsum. Some specimens from El Socorro and Boroa Norte showed the back almost totally yellowish. Froglets and adults of *E. migueli* differed strongly in their dorsal colour patterns, especially those detected in El Socorro and Boroa Norte. Formas (1977) reported three different dorsal and ventral colour patterns in specimens from the type locality (Quebrada Casanova, Mehuín). The belly pattern showed a variation ranging from almost entirely dark with small white spots to completely white. Formas (opus cit.) suggested that the colour pattern for *E. roseus* and *E. migueli* were basically the same. However, when we compared both species, very different patterns were observed. Dorsal pigmentation in *Eupsophus roseus* ranges from the typical red (*roseus*) to dark with white bars on the dorsum and limbs (tabby like) with the belly having flesh-like tones. Conversely, most *E. migueli* specimens do not present spots or bars on the legs, and the belly has reticulations in different tones and patterns, but never flesh-coloured. Moreover, *E. roseus* and *E. calcaratus* present small whitish bars in the loreal region, which are not present in *E. migueli*. Figure 2 shows four new patterns of dorsal and ventral pigmentation for specimens of *E. migueli* from new localities considered in this study.

### Reproductive strategy

A nidicolous reproductive strategy has been described for *E. altor*, *E. calcaratus*, *E. emiliopugini*, and *E. vertebralis* (Vera-Candioti et al. 2005, Núñez and Úbeda 2009, Núñez et al. 2012). The presence of calling males of *E. migueli* hidden in the moss or between roots of bushes on the banks of small streams, and the finding of newly metamorphosed frogs in the same environments in early austral spring, suggests that *E. migueli* exhibits this same strategy of larval development. At least 45 to 50 eggs can be found in each nest (ground cavity), which are cared by the male parent (Núñez et al. 2012). In mid-January 2017, a nest with 14 larvae in an advanced stage (42) of development was found on the banks of a stream in Pirén Bajo (Cuevas Personal observation). However, the male was no longer in the nest, therefore, the larvae could belong to *E. migueli* or *E. vertebralis*, considering both species have endotrophic tadpoles without a free-swimming phase (Formas 1979, Vera-Candioti et al. 2011, Núñez et al. 2012), and both species are sympatric in this area. Endotrophy has been considered an adaptive condition to the humid ground of the *Nothofagus* forest (Formas 1979, Cuevas and Cifuentes 2009) and is shared with other species that present parental care such as *Rhinoderma* spp. (Formas 2013) whose larvae are developed into the guttural sac (Formas et al. 1975).

### Habitat preferences

In the last four decades, *Nothofagus* forests of the Coastal Range (33° to 38° South) have been disturbed and destroyed to a large extent by anthropogenic activities, including agriculture and forestation (Smith-Ramírez 2005). In addition, forest fires have also caused an important loss of native forest during summer months (Cuevas and Cifuentes 2009). Subsequently, the forest matrix has changed from native to mixed, being in many cases the dominant matrix (Lara et al. 2012). All these threats have important consequences in the quality and quantity of habitat available for the frogs, and represent one of the most important causes of their decline (IUCN 2019).

**Figure 3.**
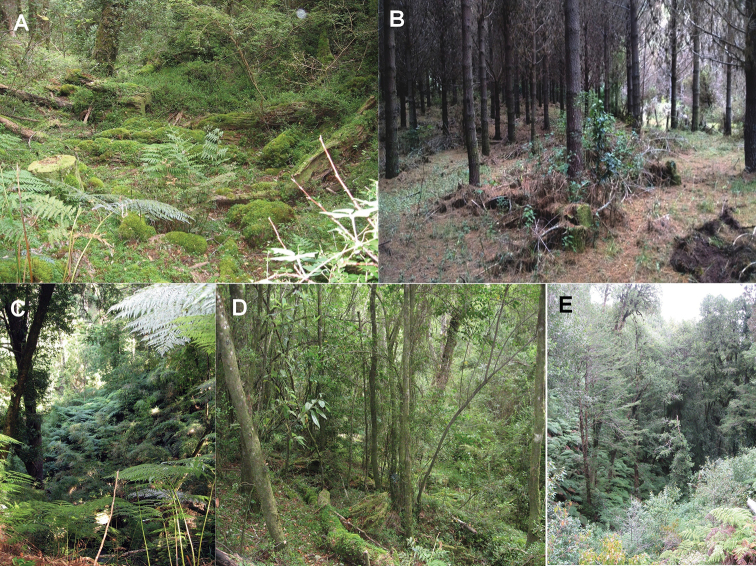
Habitat of *Eupsophus migueli***A, D** native forest with anthropogenic disturbance in Boroa Norte 1 **B** pine monoculture with undergrowth habitat of *Aristotelia chilensis* in Boroa Norte 4 **C, E** native forest in El Socorro.

In these degraded forests, the detection of *E. migueli* occurred in four different habitats as follows (see Fig. 3): a) In **ecotonal zones**, adult males were detected by their advertisement call between August and the end of October (austral spring) on the banks of small streams (1 to 1.5 m wide; 20 cm deep) surrounded by swampy areas with a lot of moss and mud. In the same habitats, froglets (5–10 mm SVL) were found camouflaged among dried leaves, moss, and small wet branches near places where adults make their nests in the mud in early spring (September) until February (exceptionally); b) In **renewal forest**, adults (males and females), pre-adults and juveniles were collected among the leaf litter, under fallen logs and rocks, both on the shore and inland forest between December and May; c) In **pine monocultures**, adults were encountered under fallen branches or logs in April, d) In **remnant native forest**, adult frogs were found among the soil litter (dry leaves) and under fallen logs. In summary, from late winter to October adults were located through their vocalizations. Then, and until late summer, adults were found only through direct searches between the litter near water courses. During autumn, individuals were found hidden in refuges under fallen logs and rocks without emitting vocalizations, being detected exclusively by active searching. These findings suggest that *E. migueli* specimens move between streams and the inner forest after the breeding season.

In the aforementioned habitat, *E. migueli* was found to be sympatric with *E. vertebralis*, *Batrachyla taeniata*, *B. leptopus*, *Pleurodema thaul* and *Rhinoderma darwinii* all along its distribution range (unpublished data). In fact, during the current study, mating calls from *B. leptopus*, *E. vertebralis* and *P. thaul* were detected; however, those differ notably from that of *E. migueli* (Penna and Veloso 1990). Lastly, *E. roseus* was also recorded in the area, although never at the same sites as *E. migueli*, suggesting that these are parapatric species (Cuevas pers. observation).

### Conservation status

Currently, *E. migueli* is categorized as “Endangered” and “Rare” by the Species Classification Regulation of the Ministry of Environment of Chile (MINSEGPRES 2008), according to criteria **B1ab (iii)** (< 5000 km^2^ presence extension) + **B2ab (iii)** (< 500 km^2^ occupancy area). The species has also been categorized as “Endangered” by the IUCN**B1ab (iii)** (IUCN 2017). In both categorizations, **B1** states that the EOO is estimated to be less than 5,000 km^2^. **B1a** states that their habitat is severely fragmented or the species is known to exist at no more than five locations, and **(iii)** states that a continued decline is observed in the area, extent and quality of habitat. Criterion **B2**, indicates an estimated area of occupancy of less than 500 km^2^, and **B2a** states that the habitat is severely fragmented, or the species is known to exist at no more than five locations, and **(iii)** establishes a continued decline observed in the area, extent and quality of habitat. Based on our surveys, both criteria are still valid regarding *E. migueli* status. The new data indicates its presence in more than five locations and new localities increase its range area from 3.223 km² (corresponding to type location) to over 200 km². Thus, the estimated values for AOO and EOOIUCN criteria was 80,000 km^2^, and 223,811 km^2^ respectively. Accordingly, *E. migueli* must remain categorized as an “Endangered” species.

On the other hand, the application of the criterion “rare species” must be revised in the case of *E. migueli*. This criterion explicitly refers to very infrequent species, and based on the surveys described here, it may have been misapplied in this case. This study verifies that *E. migueli* is a common species in the area.

**Figure 4.**
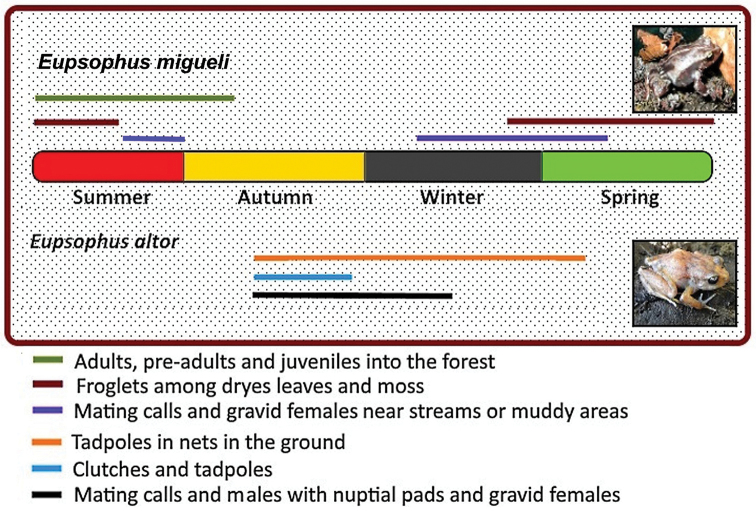
Scheme of the life history data of *E. migueli* (this paper) and *E. altor*. Data for *E. altor* were obtained from Núñez et al. (2012).

### Conclusions

The finding of additional *E. migueli* populations in small fragments within the Valdivian rainforest, and even into *Pinus* monoculture (with abundant native understory), has important implications for conservation. These findings raise questions regarding new practices for *Pinus* and *Eucalyptus* harvesting, and for vegetation management of streams, all of which are legislated in Chile. As it has been reported before, although these environments are not optimal for amphibians, they may indeed sustain some populations, as long as they have the minimal structural conditions of the undergrowth and microclimatic conditions (temperature, relative humidity, pH). However, the amphibian fauna detected in these altered environments remains in a “vulnerable” status which can be seriously affected if forest management (silviculture) interventions, such as, thinning and harvesting are not handled as “controlled disruptions” to mitigate their impacts. Disregarding these measures entails a serious risk for the amphibian diversity in the region.

Historically, the majority of the Mahuidanche Range has been covered by temperate *Nothofagus* forests (see Formas 1979, Veblen and Schlegel 1982). However, today the western slopes exhibit a high degree of human intervention (*Pinus* and *Eucalyptus* plantations, cattle raising and clearing of the native forest). Despite this, two recently described amphibians, *Alsodes norae* and *Eupsophus altor*, have been reported in the area (Cuevas 2008, Núñez et al. 2012). These species as well as *E. migueli*, *Insuetophrynus acarpicus*, *Telmatobufo australis* and in some cases *Rhinoderma darwinii* can all be found in native forest remnants. Such diversity makes the Cordillera de Mahuidanche a location of great zoogeographical and conservation interest.
